# A Parametric study on the effects of winglet cant angle on wing aerodynamics and aeroacoustics

**DOI:** 10.1038/s41598-024-84453-6

**Published:** 2025-01-09

**Authors:** Erfan Vaezi, S. Amirreza S. Madani, Amir Keshmiri

**Affiliations:** 1 Department of Aerospace Engineering, Sharif University of Technology, Azadi St., Tehran, 11155-1639 Tehran Iran; 2 Faculty of Aerospace Engineering, Delft University of Technology, Kluyverweg 1, Delft, 2629 HS South Holland The Netherlands; 3 School of Engineering, The University of Manchester, Oxford Road, Manchester, M13 9PL UK

**Keywords:** Blended Winglet, Numerical Simulation, Cant Angle, Sensitivity Analysis, Aeroacoustic Performance, Aerospace engineering, Mechanical engineering

## Abstract

The use of winglet devices is an efficient technique for enhancing aerodynamic performance. This study investigates the effects of winglet cant angles on both the aerodynamics and aeroacoustics of a commercial wing, comparing them to other significant parameters using a parametric analysis. A Full Factorial Design method is employed to generate a matrix of experiments, facilitating a detailed exploration of flow physics, with lift-to-drag ratio (L/D) and the integral of Acoustic Power Level (APL) as the primary representatives of aerodynamic and acoustic performance, respectively. The RANS formulation, along with the $k{-}\epsilon$ Realizable model and the Broadband Noise Source (BNS) model, are utilized to accurately simulate subsonic flows numerically. The study begins by examining the pressure coefficient ($C_p$) and APL distributions at various cant angles near the wingtip and root areas. The matrix of experiments is then analyzed to identify the most influential parameters based on the main effects of inputs and their two-way interactions. The results demonstrate that variations in winglet cant angle significantly alter the distribution of $C_p$ and APL along the span, particularly near the wingtip, and that cant angle strongly impacts overall performance, at times even outweighing atmospheric parameters such as pressure and temperature.

## Introduction

During the past decades, regulatory civil aviation agencies have strongly encouraged aircraft manufacturers to enhance their products’ operational efficiency^17^. Performance enhancement is able to decrease aerodynamic noise, and environmental emissions by reducing Specific Fuel Consumption (SFC)^50^. As a result, more portion of the commercial aviation market would be achieved. The main contributing parameters in this goal are such as specific fuel consumption^17^, flight range, take-off distance, climb rate, and payload capacity^6,23,29^ aerodynamics and aeroacoustic performance^20,44^, and structural enhancement^6,43,48^. To do so, one of the most straightforward manners for incrementing L/D is to increase wingspan. However, this modification imposes extra bending moments, which requires strengthening considerations for wing structure^7^ or implementing cutting-edge aircraft configurations such as truss-braced wing^25^. Moreover, it may cause potential limitations regarding airport ground operations and gate clearance requirements^17^. Also, the aerodynamic limitations imposed by high wingspan values, such as stall conditions, refuse its practical implementation in commercial aircraft^37^.

The alternative is winglet devices, which cover various aerodynamic geometries^14^. The emergence of modern winglets dates back to the 1970s when the global oil crises had significantly affected commercial airlines’ economies^17^. At that period, Whitcomb^48^ introduced a class of novel winglets at NASA Langley Research Center to reduce fuel consumption, a critical concern due to the high jet fuel price. These devices enforce more expense to design teams while relatively enhancing L/D by reducing lift-induced drag. This component usually causes 40% and 90% of total drag during cruise conditions and take-off and climb, respectively^3,13,22,23^. In terms of stability, winglets increase wingspan, which is negligible for large commercial aircraft, and enhance lateral and directional stability by increasing the vertical surfaces of aircraft^35^.

Mainly, winglet devices enhance aerodynamic performance by controlling wingtip vortices. Due to the march of crossflow from the high-pressure surface to the tip area, wingtip vortices are generated since the flow tries to replace with available air above the low-pressure surface^17^. This phenomenon causes agglomeration of vortices near the trailing edge, called wingtip vortices. This type of vortices has two primary features, (i) high velocity and (ii) low pressure, particularly within their cores^17^. Thus, winglets reduce their strength, particularly near the trailing edge^27^.

Although winglet devices provide several benefits, they adversely affect structural issues and aerodynamic deficits. Regardless of their geometry, winglets increase bending moment at the wing root, requiring additional considerations and maintenance^12^. Moreover, due to the extra skin friction, inference drag, and pressure drag, the parasite drag of wing configuration is also increased^46,47^. Consequently, the wing’s aerodynamic noise is increased in terms of both propagation and power level^44^. In light of the above, design teams should calculate changes in parasite and lift-induced drag components prior to deciding on implementing winglet devices.

Moreover, winglet devices increase the surface noise generated by the wing configuration. They not only increase acoustic noise in terms of integral and maximum values, but lead to its wider propagation downstream of the wing in similar conditions^44^. According to the literature, the noise increment in wings with blended winglets compared with simple wings is approximately 3.4%, regardless of the Mach number^44^. However, a few papers have addressed this issue in the commercial aviation compared to applications of winglet in other fields, such as wind turbine^9^ and axial fans^24^.

**Fig. 1:**
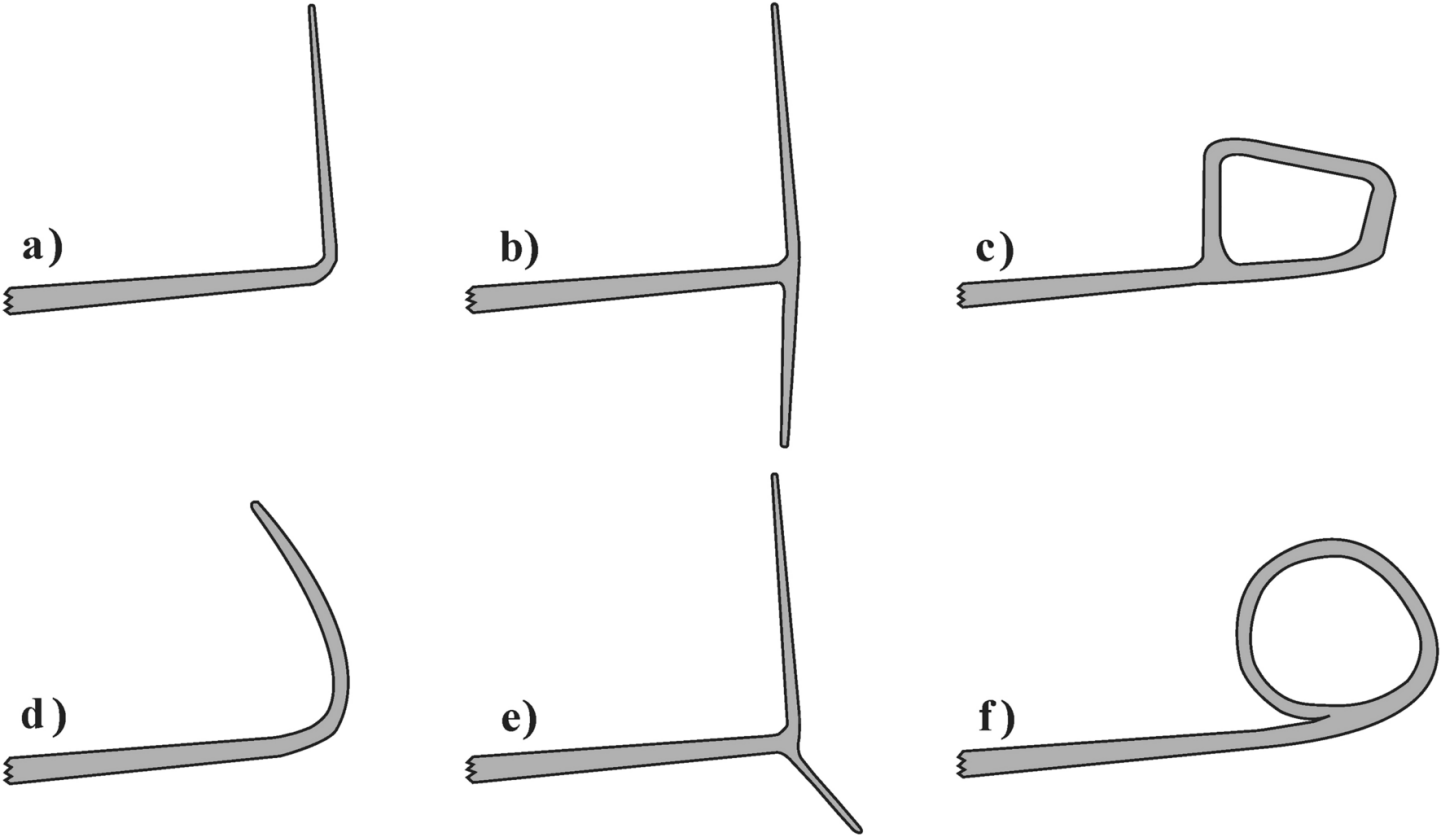
Some of the proposed winglet configurations; (**a**) blended, (**b**) wingtip fence, (**c**) spiroid, (**d**) sharklet, (**e**) whitcomb, and (**f**) circular.

Up to now, various conceptual designs of winglets have been proposed with different geometric properties and spatial angles. Figure 1 depicts some of the proposed winglets, most of which are classified as fixed/passive, providing optimum operation in one standard on-design point during a flight. As a result, the winglets’ efficiency is maximum at a single flight condition, while it may not be optimum during other flight states. Therefore, using fixed designs in off-design points results in adverse consequences such as extra surface and smaller chord at the wingtip resulting in profile drag increment and earlier flow separation and stalling, respectively^23,28,33,38^.

As discussed, using winglet devices in off-design points results in less aerodynamic amelioration compared to both on-design points and simple wings^34^. One practical idea to address this issue is to design rotary winglets which could experience all possible states between planar configurations and vertical layouts. This design is more cost-efficient compared to the available alternatives since it imposes minimum additional costs and mechanisms and preserves the integrity of wing configurations^10,41^.

Recent research has examined the effects of winglet cant angle on aircraft performance. In this context, the commercial Computational Fluid Dynamics (CFD) code. Fluent was used by Helal et al.^18^ to investigate the effects of cant angle on a wing based on NACA 65-218 airfoil. Winglets were found to enhance *L*/*D* in a range of 6% to 15%. Furthermore, they presented tables providing optimum cant angles for every angle of attack. Guerrero et al.^17^ proposed using variable cant angle winglets, in which aircraft can achieve higher performance in terms of induced drag reduction in each phase of a flight. Moreover, they investigated the effect of winglet cant angle and sweep angle on the performance of a benchmark wing at Mach numbers of 0.3 and 0.84. The results illustrated that an optimal selection of winglet cant angle can enhance the aerodynamic performance.

Soreshjani and Jahangirian^42^ proposed a fast and efficient method for winglet design optimizations. Their proposed method is superior in terms of accuracy and convergence speed compared to current methods; afterward, they performed a winglet design optimization problem for the DLR-F6 wing. As a result, the optimal drag coefficient of the winglet was reduced by 9.19% compared to the initial wing; however, the lift coefficient was unchanged. Furthermore, they studied the effect of design parameters in the optimization process and showed that the angle of attack, the twist angle, and the winglet length significantly affect the winglet design.

Seshaiah et al.^39^ modeled various winglets in terms of different cant angles, including $35^\circ$, $65^\circ$, and $90^\circ$; They simulated winglets computationally using Ansys. They noticed that wings with winglets achieved higher L/D. In Kaygan’s paper^21^, a morphing winglet concept has been computationally investigated utilizing AVL (Athena Vortex Lattice) Method. The aerodynamic benefits of this concept in various flight conditions are also demonstrated by numerical investigations on values of twist angle ($-10^\circ$ - $10^\circ$) and dihedral angle ($-90^\circ$ - $90^\circ$). As a result, the highest L/D was achieved at a twist angle of $-5^\circ$ and a dihedral angle of $0^\circ$.

According to the existing literature, available papers have discussed the aerodynamic effects of the cant angle without recognizing and involving other effective parameters^17,18,39^, while a comprehensive optimization of the performance of the winglets for points of non-design requires accurate consideration of not only the cant angle but also the atmospheric and operational conditions.

The primary motivation of this work is to conduct a comparative investigation in order to find out the effectiveness of the cant angle compared to pressure, temperature, Mach number, and angle of attack. For this purpose, a multidisciplinary approach is used to explore the dependency of selected performance parameters. This novel approach leads to a comprehensive understanding of aerodynamic and acoustic parameters in addition to the available articles on stability analysis^35^ and structural considerations^7^.

**Fig. 2:**
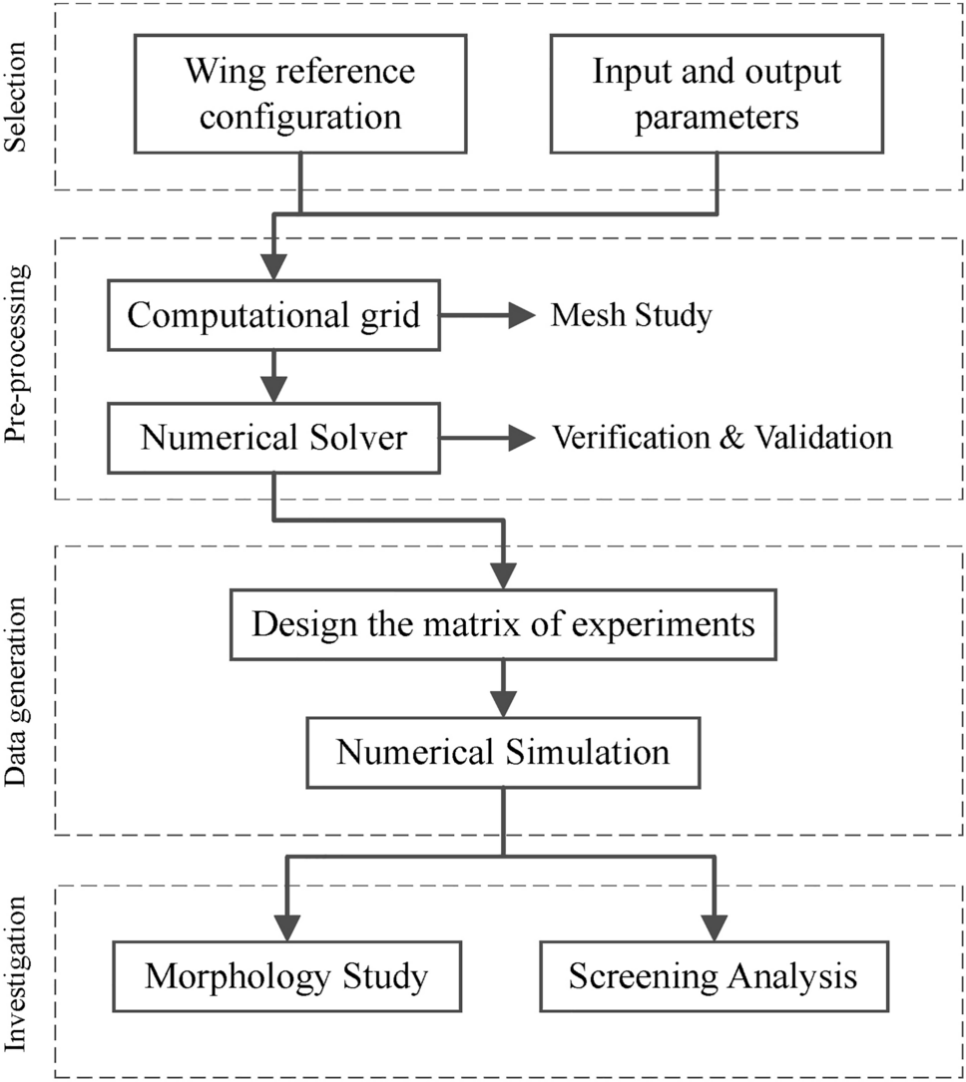
Roadmap of the current research work.

Figure 2 shows the research roadmap that includes a matrix of experiments initially developed using a full-resolution Full Factorial Design to generate standard datasets. A CFD solver, consisting of RANS (Reynolds-Averaged Navier-Stokes) formulation and $k-\epsilon$ Realizable turbulence model, is used to extract L/D and integral of APL (Acoustic Power Level) parameters as representatives of aerodynamic and acoustic performance. Prior to analyzing datasets, the pre-processing phase is conducted to check datasets regarding the existence of multi-value instances by elimination. The investigation phase of this research consists of the following two primary sections:

- **Morphology study:** Studying aerodynamic and acoustic effects of cant angle using $C_P$ and APL distribution along sections near the wingtip and root.
- **Screening:** By analysis of the obtained matrix of experiments using MiniTab software^1,26^ the main effects of the factors and their two-way interactions are assessed. Hence, the most effective terms are recognized in terms of L/D and integral of APL.

This research distinguishes itself by providing a comprehensive parametric investigation into the effects of winglet cant angle on both aerodynamic and aeroacoustic performance, considering its comparative significance relative to atmospheric and operational parameters such as pressure, temperature, Mach number, and angle of attack. Using a systematic Full Factorial Design, the study explores the main effects and two-way interactions of these variables, enabling a robust evaluation of their influence on lift-to-drag ratio (*L*/*D*) and acoustic power level (APL). Unlike prior works that focused predominantly on single-flight conditions or isolated parameters, this research incorporates detailed CFD simulations to uncover critical dependencies and interactions. The dual emphasis on aerodynamics and acoustics, particularly under varying flight states, offers a more holistic understanding of performance optimization, addressing gaps in prior literature regarding winglet design and effectiveness.

## Methodology

### Baseline platform

**Fig. 3:**
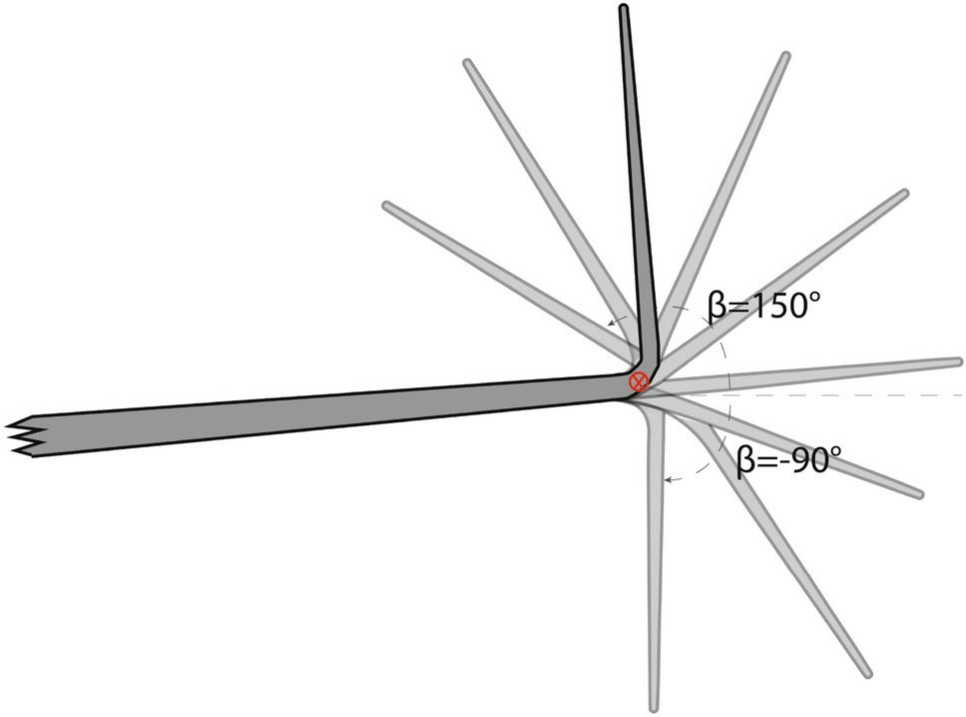
Front view of eight blended wings with different cant angle.

Aerospace engineers try to enhance aircraft performance by implementing lift augmentation and drag reduction devices to design aerodynamically-efficient wings^37^. Winglet devices are a typical design pattern for aircraft designers^11^. Thanks to its simplicity, the most common design for commercial aircraft is the blended type^34^. As shown in Figure 3, zero cant angle is considered parallel to wingspan and rotation direction is assumed counter-clockwise.

The wing configuration of the Boeing 737-800 was selected as the baseline platform. Table 1 provides the geometric properties of the inner and outer parts of the wing.

**Table 1:** Geometric characteristics of the baseline wing

Characteristic	Inner Wing	Outer Wing
Root airfoil name	b737a-il	b737b-il
Tip airfoil name	b737b-il	b737c-il
Root chord [m]	7.61	3.81
Tip chord [m]	3.81	1.36
Taper ratio	0.5	0.35
Half-span [m]	5.5	11.5
Dihedral angle [$^\circ$]	9	6

### Numerical solver

This study uses Ansys Fluent 21 R1 to simulate turbulent compressible flow to provide a detailed understanding of the physics and aerodynamic and acoustic parameters^15^. For this purpose, CATIA and ICEM CFD are used to design the 3D geometry of the wing and generate the numerical mesh, respectively. The numerical solver is set based on the following assumptions:

- The flow is steady and compressible
- The effects of wall roughness and gravity are not taken into account
- Air is an ideal gas with a temperature-dependent viscosity

#### Computational grid

**Fig. 4:**
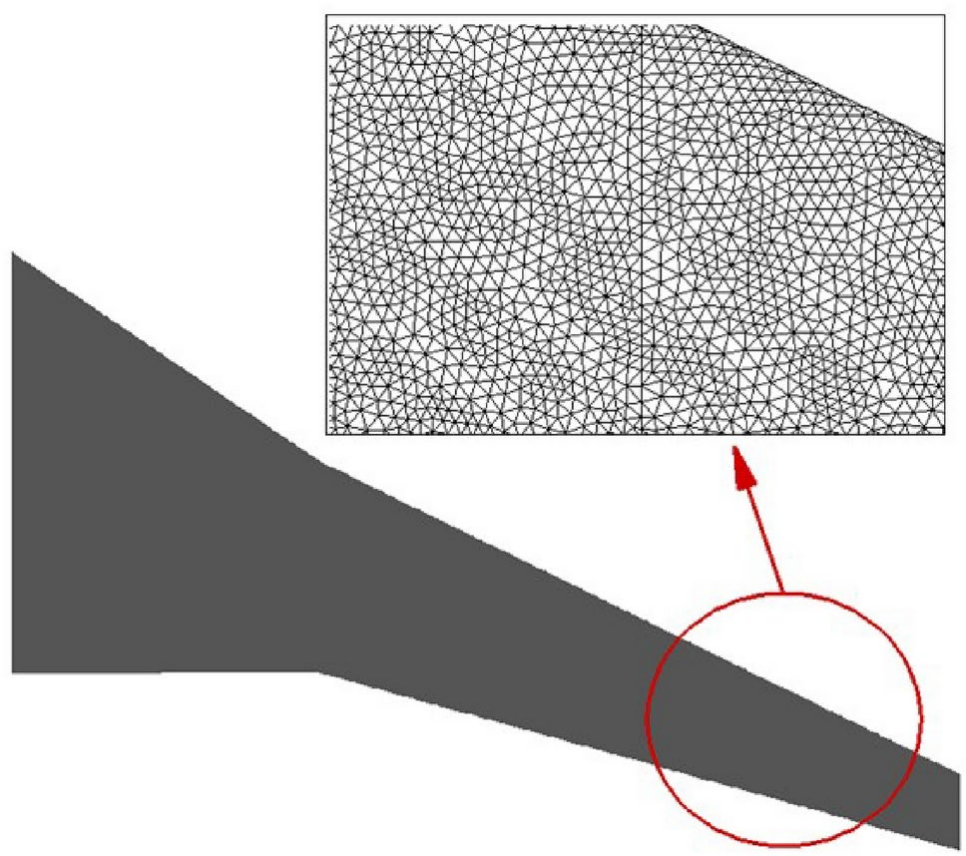
Hybrid computational grid on wingtip surface.

**Fig. 5:**
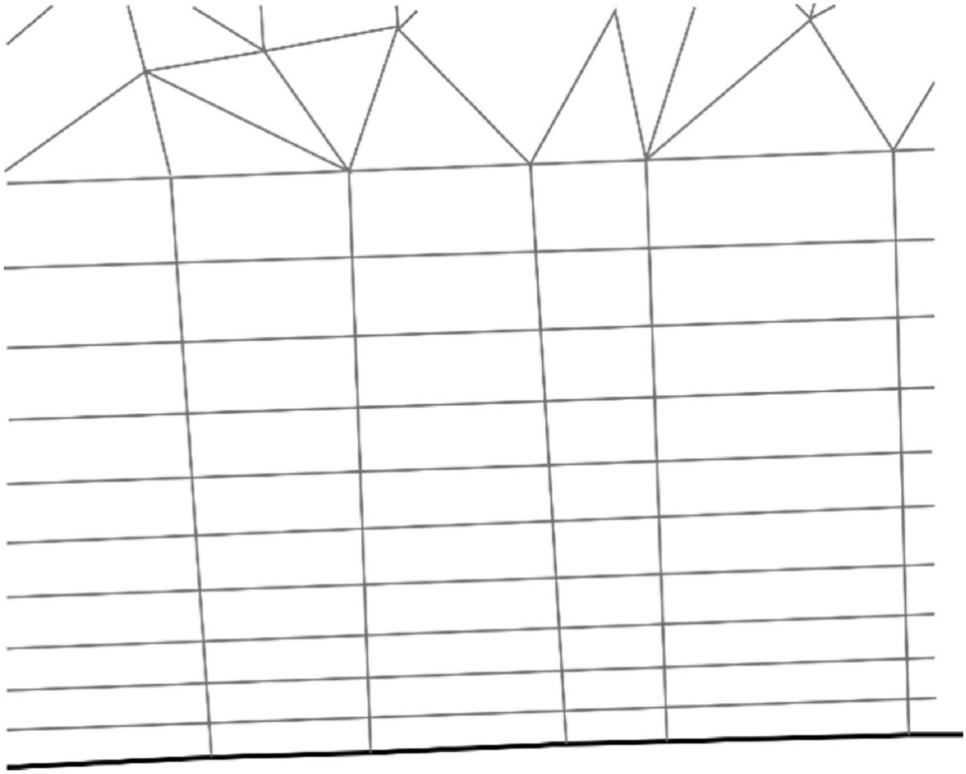
Hybrid computational grid within boundary layer.

The hybrid mesh type is selected to discretize the control volume, which is structured within the wing’s boundary layer area and unstructured in the rest of the control volume. Figures 4 and 5 illustrate the computational grid on the wing surface and the boundary layer area. Table 2 details the final computational grid used for the numerical simulations of this study.

**Table 2:** Details of the computational grid

Parameter	Value
y+	1.1
Height Ratio	1.1
Number of Layers	10
Total Number of Elements	2,350,000

#### Fluid flow equations

The three-dimensional formulation of RANS method and $k-\epsilon$ realizable turbulence model are used to simulate the flow field and turbulence effects, respectively. This choice is made due to the flow complexity caused by different vortices such as tip vortex, wake, and wingtip around the wing^45^; In accordance with the literature, this is a reliable model which can calculate a variety of complex flows, including boundary layer and separation based upon validating against experimental results^5^. In this system of equations, the eddy viscosity is computed as

1 $$\begin{aligned} \mu _t=\rho C_{\mu } \frac{k^2}{\epsilon } \end{aligned}$$

where $C_{\mu }$ is calculated as

2 $$\begin{aligned} C_{\mu }=\frac{1}{A_0+A_s\frac{kU^*}{\epsilon }} \end{aligned}$$

where $U^*$ is computed as

3 $$\begin{aligned} U^*=\sqrt{S_{ij}S_{ij}+\widetilde{\Omega }_{ij}\widetilde{\Omega }_{ij}} \end{aligned}$$

where

4 $$\begin{aligned} & \widetilde{\Omega }_{ij}=\Omega _{ij}-2\epsilon _{ijk}\omega _k \end{aligned}$$

5 $$\begin{aligned} & \Omega _{ij}=\overline{\Omega }_{ij}-\epsilon _{ijk}\omega _k \end{aligned}$$

where $\overline{\Omega }_{ij}$ is the mean rate-of-rotation tensor viewed in a rotating reference frame with the angular velocity $\omega _k$. The model constants $A_0$ and $A_s$ are given by

6 $$\begin{aligned} A_0= & 4.04 \end{aligned}$$

7 $$\begin{aligned} A_s= & \sqrt{6}\cos \phi \end{aligned}$$

where

8 $$\begin{aligned} \phi= & \frac{1}{3}\cos ^{-1}(\sqrt{6}W) \end{aligned}$$

9 $$\begin{aligned} W= & \frac{S_{ij}S_{jk}S_{ki}}{\widetilde{S}^3} \end{aligned}$$

10 $$\begin{aligned} \widetilde{S}= & \sqrt{S_{ij}S_{ij}} \end{aligned}$$

11 $$\begin{aligned} S_{ij}= & \frac{1}{2}\left( \frac{\partial u_j}{\partial x_i} + \frac{\partial u_i}{\partial x_j}\right) \end{aligned}$$

#### Acoustic analogy

The steady fluid flow acoustic field is simulated using the Broadband Noise Source (BNS) model. This method is based on Proudman and Curle’s boundary layer models, which consider the effects of quadrupole and dipole sources, respectively^51^. Proudman employed Lighthill theory to model the generated noise as homogeneous isotropic turbulence and presented an analytical method for determining acoustic power per unit volume based on statistical data^36^. The generated turbulent noise owing to flow separation and turbulent fluctuations within the boundary layer could be calculated using the Proudman model.

The Curle’s boundary layer model is based on the Curle integral, where the radiated acoustic pressure is derived as a function of the fluctuating surface pressure of rigid body surfaces^8^. This model can measure noise induced by pressure fluctuations on the surface. In this study, the generated noise is mainly caused by flow separation and pressure fluctuations, which are investigated using the BNS model. In this context, Mohamud and Johnson^31^ presented an analytical relation for determining the acoustic power per unit ($P_A$) as Equation 12.

12 $$\begin{aligned} P_A=c\rho _0 \left( \frac{u^3}{l}\right) \left( \frac{u}{a_0} \right) ^5 \end{aligned}$$

where the variables $\rho _0$, $a_0$, *l* and *u* are density, speed of sound, turbulent length scales and turbulent velocity, respectively, where the variable *u* is calculated from Equation 13 and *k* represents the turbulent kinetic energy.

13 $$\begin{aligned} u^2 = \frac{2}{3}k \end{aligned}$$

*c* is also a constant value, which Proudmann obtained 13.5, and Neise and Koopmann obtained 37.5^32^. Fluent can provide the acoustic power variable in [dB], which is extracted from Equation 14.

14 $$\begin{aligned} L_p = 10\log \left( \frac{P_A}{P_{ref}}\right) \end{aligned}$$

where $P_{ref}$ is the reference acoustic power which is considered as $10^{-12}$ [W]^51^.

#### Boundary conditions

**Fig. 6:**
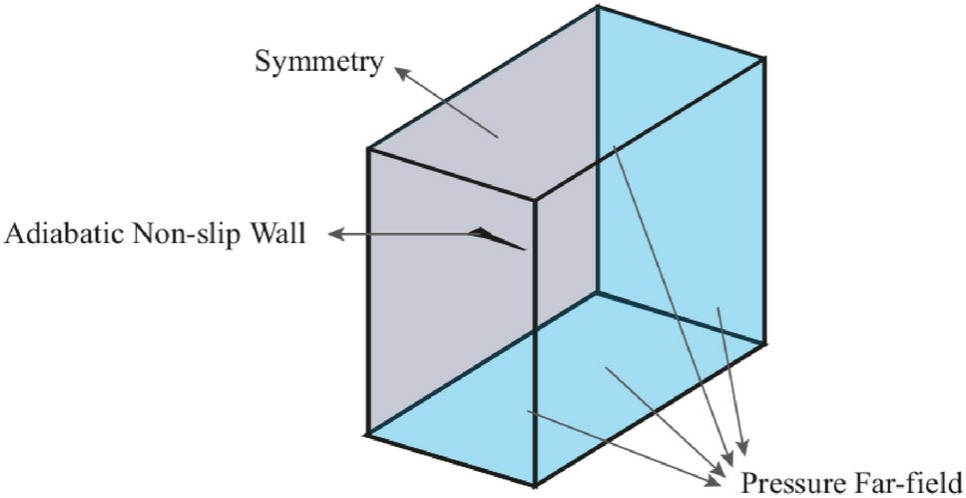
Defined control volume and applied boundary conditions.

Figure 6demonstrates applied boundary conditions to the control volume. Pressure far-field boundary condition has been applied for the inlet boundaries of the control volume. Also, No-slip, adiabatic wall was applied for wing surfaces, and the symmetric condition was selected for the side boundary of the domain.

#### Numerical scheme

The power-law scheme examines kinetic energy and turbulent dissipation rate equations in the applied numerical solver. To calculate changes in the separation function, the first-order method, and for the spatial discretization of the gradient, the least-squares cell-based method is implemented, which has a suitable performance for calculating the gradient of the acoustic field. Flow field equations are discretized by the second-order central and pseudo-transient formulation methods.

### Full factorial design

Statistical methods, such as factorial design, are reliable tools for investigating and optimizing effective factors in different processes, particularly for defining the matrix of experiments^30^. In the current study, the relevant factors are divided into two types, consisting of controllable and uncontrollable. The controllable factors are pressure, temperature, Mach number, angle of attack, and cant angle. The uncontrollable ones are mainly caused by the lack of either measuring tools or accuracy; turbulence characteristics, solver accuracy, and spatial discretization errors mainly cause uncontrollable factors in this research work. The full factorial design provides the highest resolution and would enable the study of the main effects and their two-way interactions, albeit at a cost of experiments and/or simulations^4,40^. The number of experiments in this method is calculated according to $N_E = L^f$^16^, where $N_E$, *L*, and *f* are the number of experiments, levels, and factors in the matrix of experiments.

In order to define the matrix of experiments, the range of input factors must be defined. Table 3 shows the inputs’ label, minimum, and maximum values. The detailed matrix of experiments is provided in Appendix A.

**Table 3:** Details of input factors in the matrix of experiments

Factor	Unit	Label	Range
Pressure	[kPa]	A	$[26.5,\,101.3]$
Temperature	[K]	B	$[224,\,300]$
Mach Number	[-]	C	$[0.3,\,0.8]$
Angle of Attack	[$^\circ$]	D	$[0,\,20]$
Cant Angle	[$^\circ$]	E	$[-90,\,150]$

## Validation and verification

Since there is no open dataset available in terms of aerodynamic and acoustic performance of the baseline platform, the present numerical solver is validated using different metrics, including mesh study, solver assessment, and range comparison as described below:

- **Mesh study:** To check the sensitivity of grids in terms of lift and drag coefficients.
- **Solver assessment:** To assess the implemented solver against previous research papers.
- **Range comparison:** To compare a secondary parameter with the previous results.

### Mesh study

**Fig. 7:**
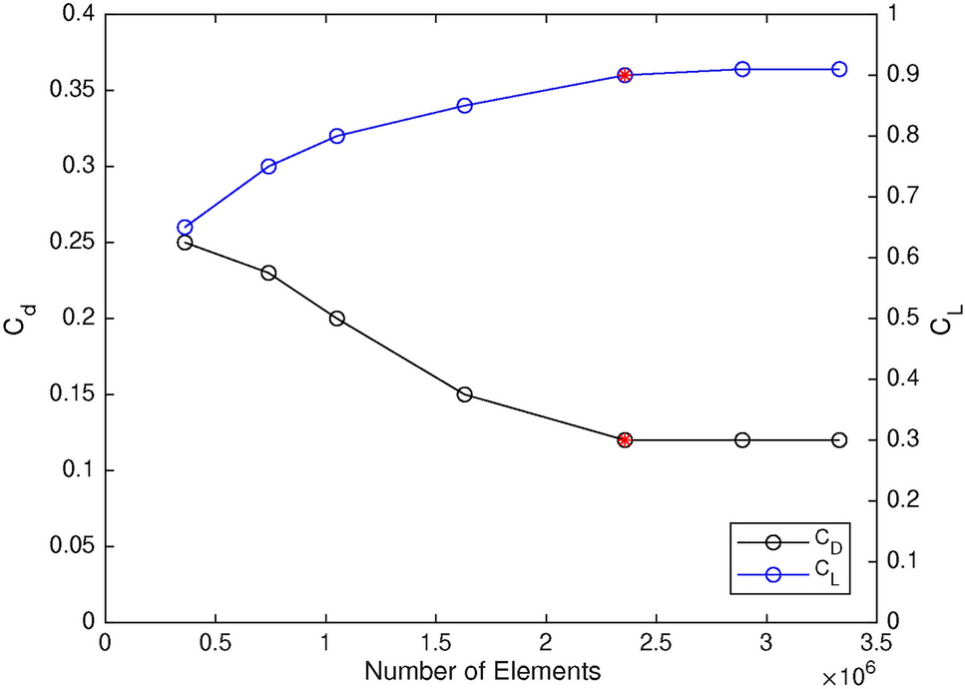
Mesh study results in terms of lift and drag coefficients.

In order to find the optimum grid size, a mesh study is conducted by calculating lift and drag coefficients for different numbers of elements. As shown in Figure 7, seven different meshes are examined, ranging from 360,000 to 3,400,000 elements. The convergence criterion is defined based on both aerodynamic ($C_L$ and $C_D$) and acoustic (integral of APL) parameters. Each simulation is stopped after simultaneous converged values for the above parameters in at least 200 iterations. Considering both accuracy and computational cost, the 2,350,000 elements mesh was deemed is the optimum setup.

### Solver assessment

In order to verify the numerical simulations, the solver’s features are compared with the ones used in some papers in the field of winglet studies. To do so, the solvers are compared in terms of case studies, grid type, turbulence model, and governing equations, as shown in Table 4. According to the table, the framework of the applied solver is compatible with the general features of utilized solvers in recent papers.

**Table 4:** Comparison of applied numerical solver with previous papers

Research	Configuration	Grid type	Applied Numerical Solver	L/D increment
Current research	Aircraft wing	Hybrid	RANS; $k-\epsilon$ Realizable	14.44 %
Gavrilović et al.^14^	Aircraft wing	Unstructured	RANS; $k-\epsilon$ Realizable	14.29 %
Vaezi et al.^44^	Aircraft wing	Hybrid	RANS; $k-\omega$ SST	14.51 %
Panagiotou et al.^35^	Aircraft wing	Hybrid	RANS; Spalart-Allmaras	15.37 %
Guerrero et al.^17^	Aircraft wing	Hybrid	RANS; $k-\omega$ SST	Not applicable
Jiang et al.^19^	Turbine blade	Structured	RANS; $k-\omega$ Standard	Not applicable
Ye et al.^49^	Axial fan	Hybrid	RANS; $k-\epsilon$ Realizable	Not applicable

### Range comparison

The accuracy of the solver is evaluated with four papers in terms of flight range enhancement. The selected papers have investigated the same compressible, turbulent fluid flow regime and similar winglet devices. In order to calculate flight range, Breguet equation is used^2^. Note that adding winglet devices only affects $C_L$ and $C_D$ values and other parameters remain constant.

**Fig. 8:**
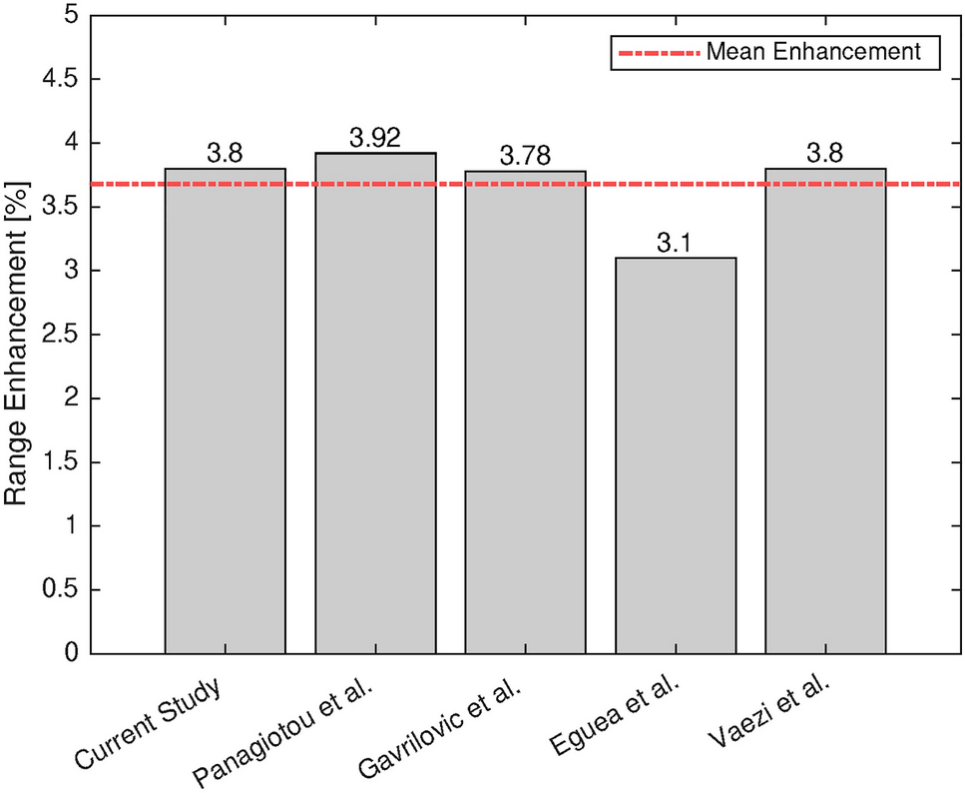
Range increment of blended winglet in current and previous works. Mentioned research in the figure: Panagiotou et al.^34^, Gavrilovic et al.^14^, Eguea et al.^10^, and Vaezi et al.^44^.

Figure 8shows the range enhancement of the blended winglet at the cant angle of $90^\circ$ compared to the simple wing for five different studies. According to Figure 8, the blended design at the cant angle of $90^\circ$ enhances flight range, ranging from 3.1% to 3.9%, which approves the work results in range analysis. Since calculating correct force coefficients requires accurate physics of flow, particularly pressure and temperature distributions, it is concluded that the present solver can obtain accurate performance parameters, which is the main objective of this paper.

## Results and discussion

This section presents the results from two perspectives: the morphology study and the comparative analysis. The morphology study focuses on the flow structure around the wing configuration, while the comparative analysis evaluates the effectiveness of the input parameters, with a particular emphasis on the winglet cant angle. Table 5 provides the maximum, minimum, and range of key performance indicators derived from the numerical simulations, specifically the lift-to-drag ratio (L/D) and the integral of Acoustic Power Level (APL). As shown in the table, the range of L/D extends from $2.23 \times 10^0$ to $1.79 \times 10^1$, while the APL ranges from $1.11 \times 10^4$ [*dB*] to $5.81 \times 10^3$ [*dB*]. These ranges capture the full spectrum of operational conditions analyzed in the study, ensuring that the results are applicable to various phases of flight.

**Table 5:** Maximum, minimum, and range of L/D and Integral of APL

Parameter	Maximum	Minimum	Range
L/D	$1.79\times 10^1$	$2.23\times 10^0$	$1.56\times 10^1$
Integral of APL	$5.81\times 10^3$	$1.11\times 10^4$	$5.30\times 10^3$

### Preliminary study

Winglet devices have a direct impact on both aerodynamics and acoustics as they modify the flow structure around the wing configuration. Consequently, performance coefficients change due to variations in pressure distribution, particularly near the wingtip area. This section explores the local and overall effects of varying winglet cant angles under constant atmospheric and operational conditions. Specifically, the distribution of the pressure coefficient and acoustic power level is analyzed at zero angle of attack for sections near both the wingtip and root areas. A comparative study of these parameters is presented in the following analysis.

#### Flow morphology

**Fig. 9:**
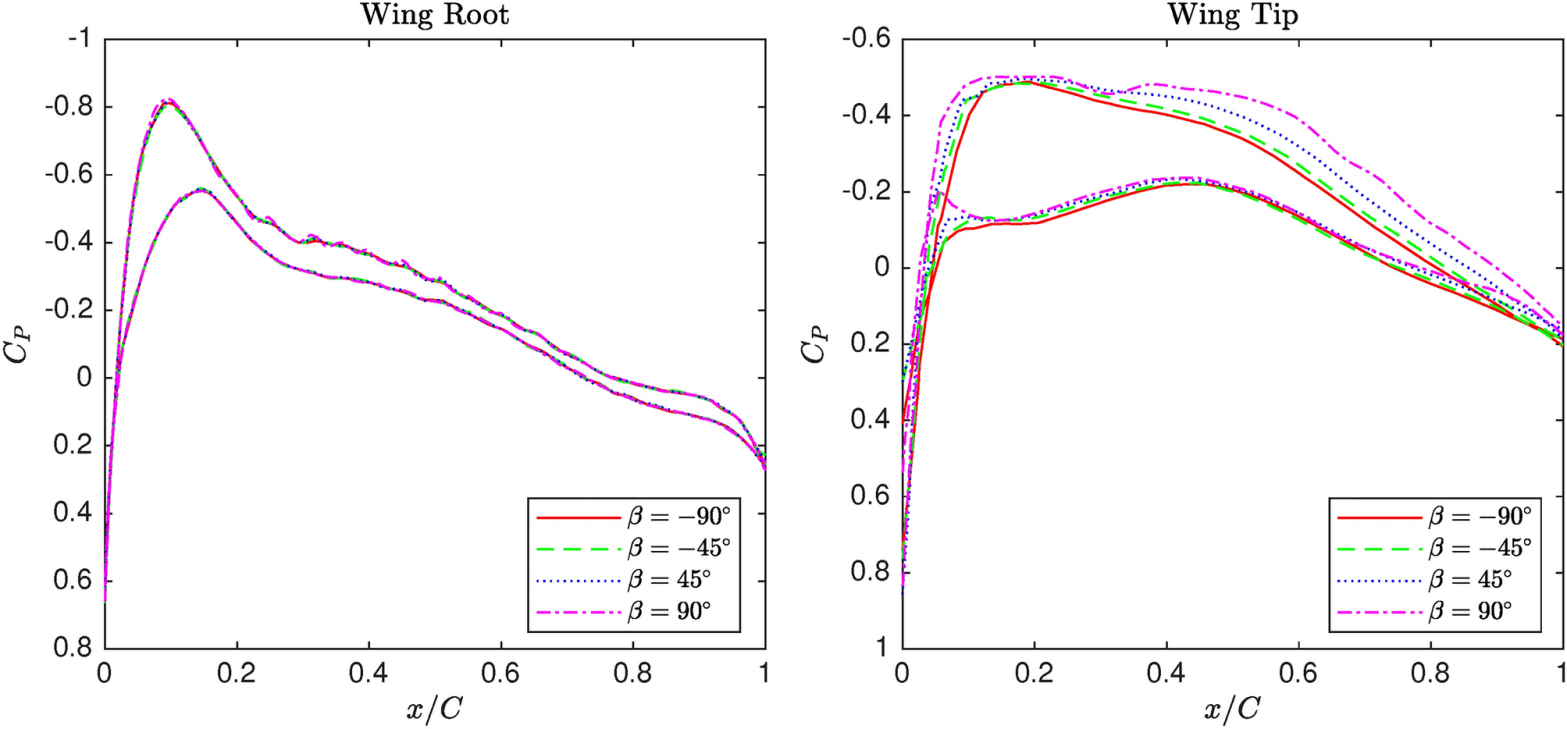
$C_P$ distribution of the blended wing for various winglet cant angles at the section near wing root and wingtip.

In this section, the fluctuations in the static pressure coefficient caused by changing the cant angle from $-90^\circ$ to $90^\circ$ are discussed. Figure 9 shows the $C_P$ distributions along two cross-sections near the wingtip and root areas at $0^\circ$ angle of attack, positioned 2.0 and 16.8 meters away from the symmetric plane. According to the figures, although the cant angle varies significantly from $-90^\circ$ to $90^\circ$, the $C_P$ distribution remains similar in general shape across the sections. However, the cant angle changes cause substantial $C_P$ fluctuations near the wingtip, while the effects on the $C_P$ diagrams are negligible near the wing root. Furthermore, the effectiveness of the cant angle increases gradually from the leading edge to the trailing edge in the wingtip sections, indicating stronger sensitivity to cant angle in this region.

**Fig. 10:**
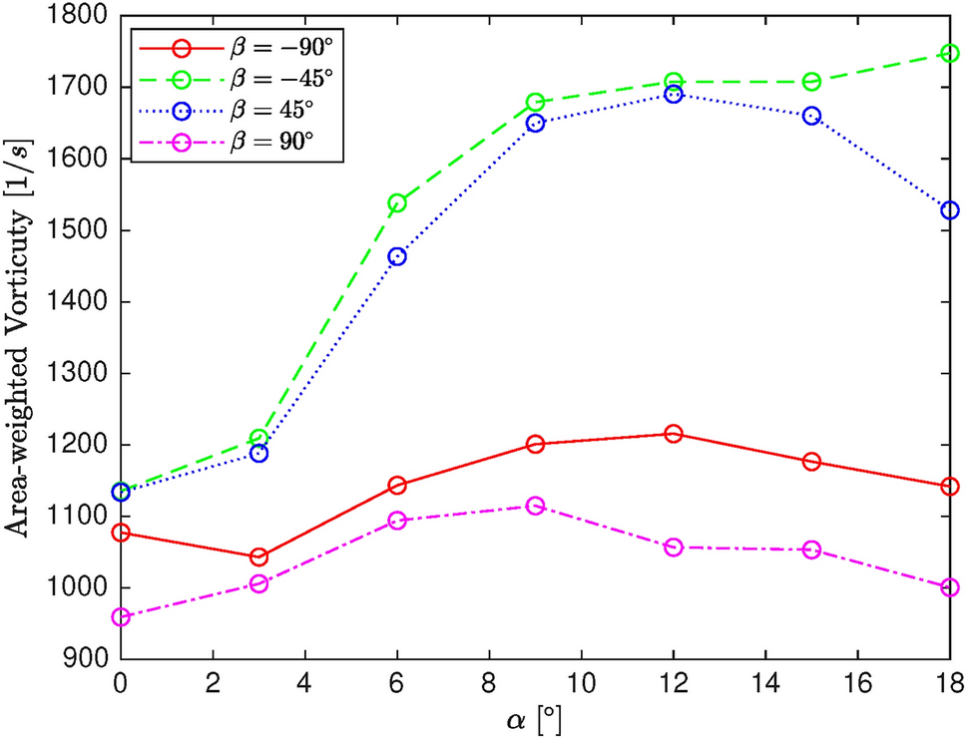
Area-weighted vorticity at wing tip for various winglet cant angles.

Figure 10 plots the area-weighted average of vorticity as a function of angle of attack for several cant angles at the wingtip location. The curves corresponding to $\beta =\pm 90^{\circ }$ are lower than those of $\beta =\pm 45^{\circ }$, indicating lower vorticity for higher cant angles. All curves exhibit a local increase in vorticity as the angle of attack increases from $\alpha =0^{\circ }$ to $\alpha =15^{\circ }$, followed by a local decrease. However, for $\beta =45^{\circ }$, the vorticity drop is less significant due to a reduction in the wetted area at higher angles of attack. In general, the configurations with $\beta =90^{\circ }$ and $\beta =-45^{\circ }$ demonstrate the best and worst performance, respectively, in terms of area-weighted vorticity. This vorticity behavior is critical for predicting aerodynamic stability and vortex strength under different flight conditions.

#### Aeroacoustic analysis

In this section, the impact of rotating winglets on the acoustic power level, representing aeroacoustic performance, is examined under similar conditions. Unlike in the previous section, the distributions on the upper and lower surfaces are shown separately, as more pronounced fluctuations are observed compared to those related to static pressure distribution.

**Fig. 11:**
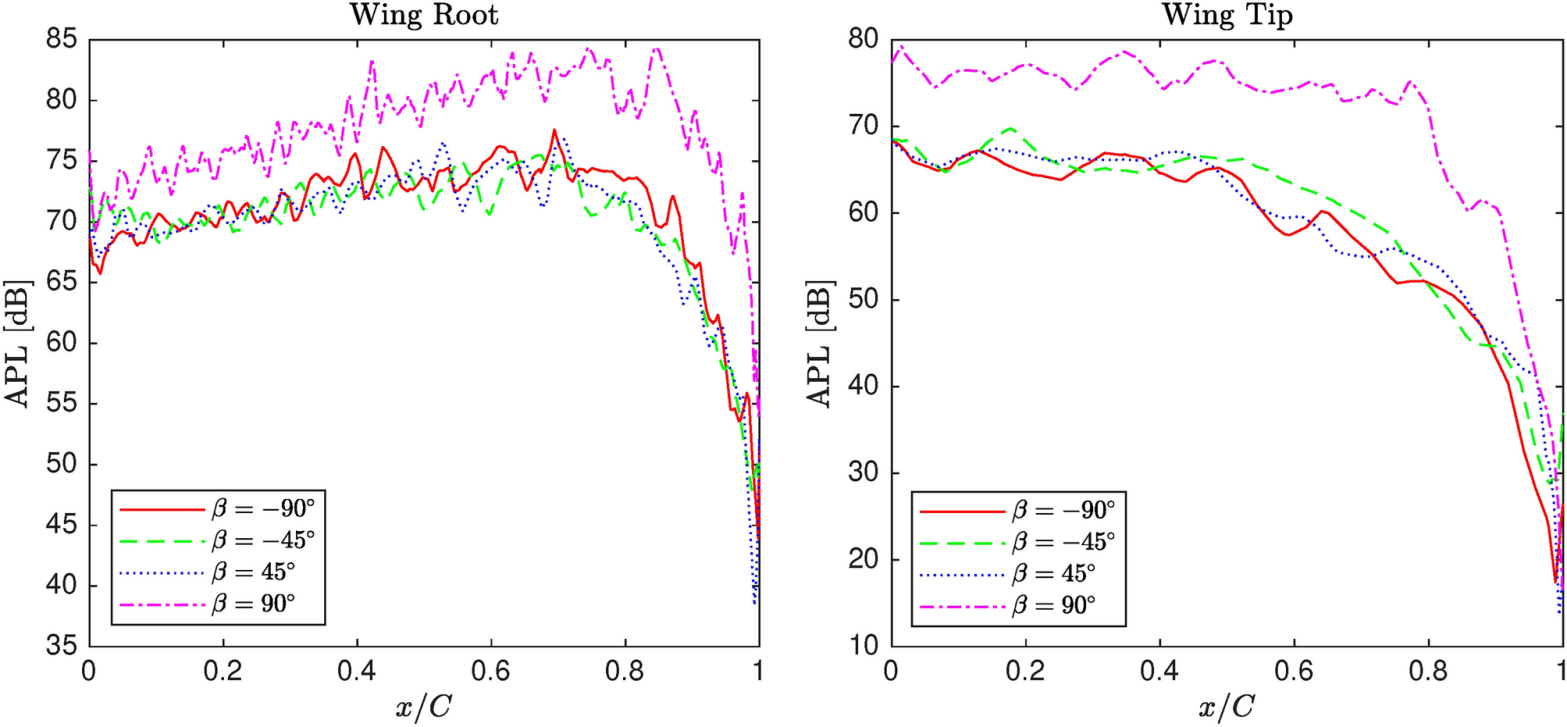
APL distribution of the blended wing for various winglet cant angles at the upper section near wing root and wingtip.

Figure 11 illustrates the acoustic power level distribution on the upper and lower surfaces of the wing near the root for various cant angles between $-90^\circ$ and $90^\circ$ at $0^\circ$ angle of attack. The effect of cant angle changes from $-90^\circ$ to $45^\circ$ is quite similar, but increasing the cant angle from $45^\circ$ to $90^\circ$ leads to a consistent rise in the acoustic power level. Additionally, the fluctuations observed on the upper surface are more pronounced than those on the lower surface, where the curves are smoother. The figures also show a significant reduction in acoustic power level in the last 20% of the half-span toward the wing root, primarily due to the decrease in flow momentum on the wing surface.

**Fig. 12:**
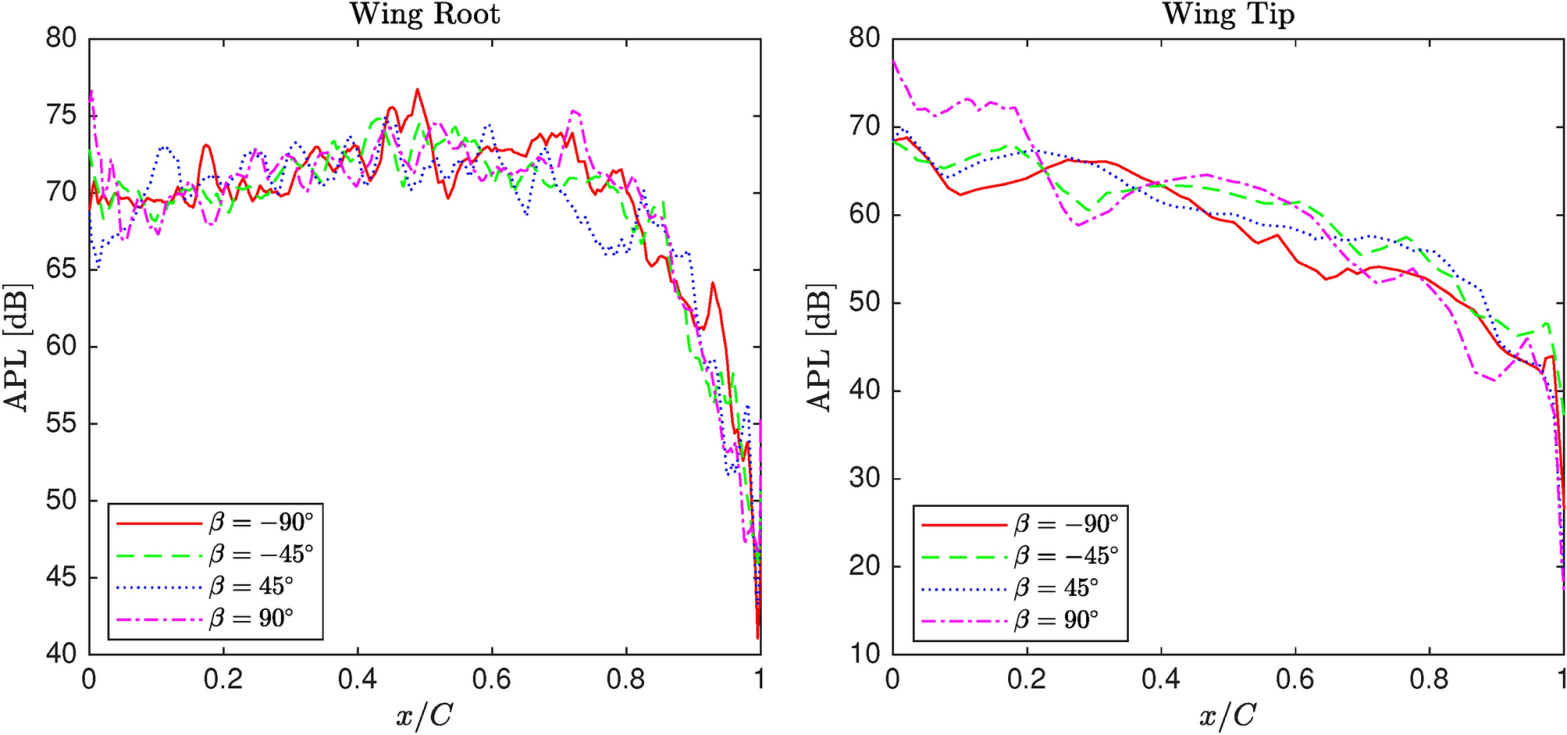
APL distribution of the blended wing for various winglet cant angles at the lower section near wing root and wingtip.

Figure 12 depicts the acoustic power level distribution on the upper and lower surfaces of the wing near the tip for various winglet cant angles between $-90^\circ$ and $90^\circ$ at $0^\circ$ angle of attack. Compared to Figure 11, the amplitude of oscillations on both upper surfaces increases for all cant angles, indicating an amplified effect of cant angle variation. On the lower surface, the curvature remains smooth, although low-amplitude fluctuations are more evident. This distinction between the upper and lower surfaces suggests a stronger aerodynamic sensitivity in the upper regions of the wing.

### Sensitivity analysis

In this section, a screening analysis is conducted on the matrix of experiments using MATLAB. The primary objective is to provide a comprehensive evaluation of the main effects of the input factors and their two-way interactions. Main effects measure the independent effectiveness of each input, while two-way interactions represent the combined influence of each pair of inputs. To achieve this, the performance of the screening models for both L/D and the integral of APL, as outputs, is evaluated based on their R-squared values. Residual plots-including normal probability plots, residuals versus fitted values, and residual histograms-are examined for each output independently. The screening results are then presented through main effects and two-way interaction plots. Finally, the associated Pareto charts are compared to identify the most and least influential factors.

#### Model performance assessment

To evaluate the accuracy of the screening models, overall, adjusted, and predicted R-squared values were calculated for each model. As shown in Table 6, models achieve a minimum accuracy of 95%.

**Table 6:** Accuracy of the proposed screening models based on R-Squared values.

Parameter	L/D	Integral of APL
Overall R-Squared	96.71%	99.79%
Adjusted R-Squared	96.08%	99.68%
Predicted R-Squared	95.02%	99.47%

**Fig. 13:**
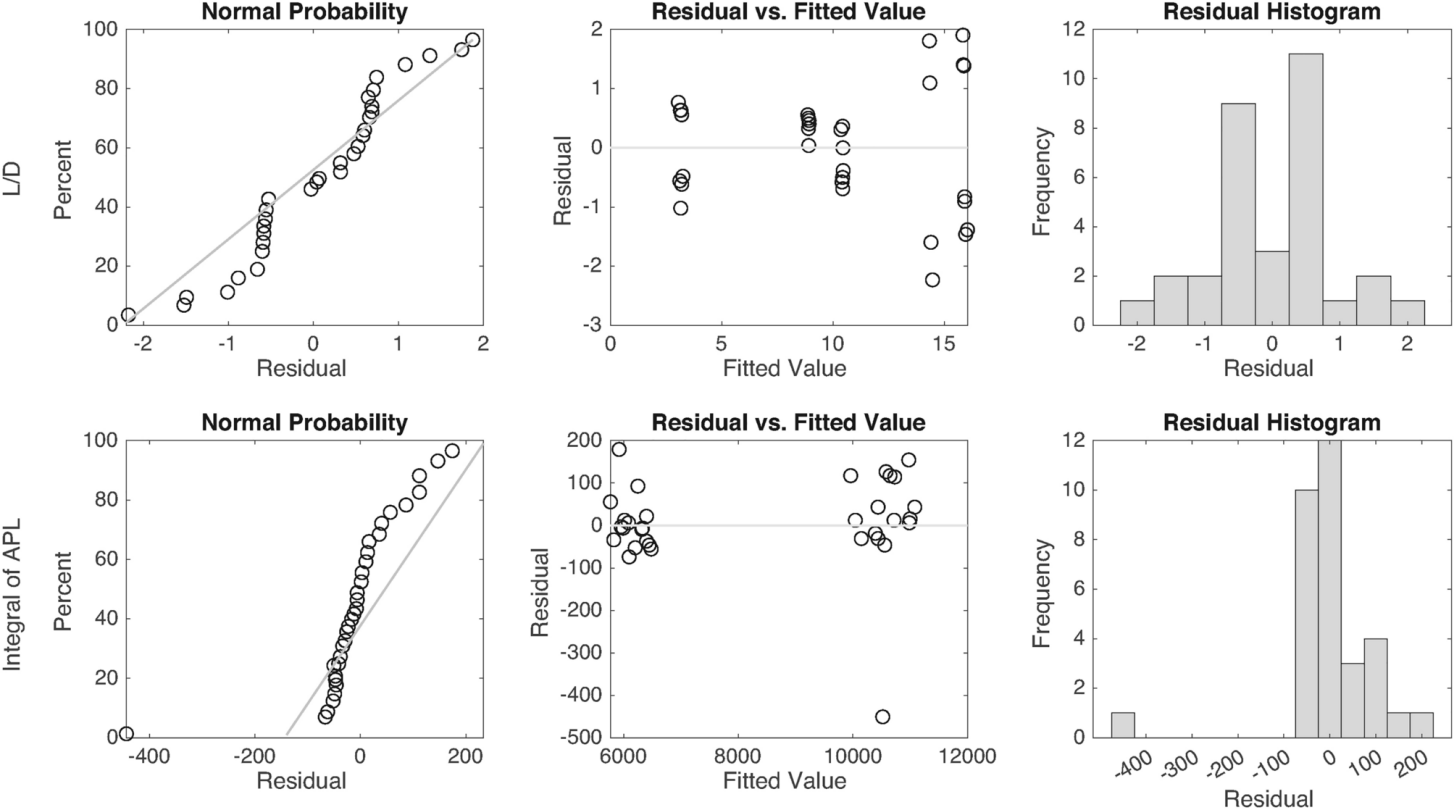
L/D and integral of APL screening models’ residuals.

Figure 13presents the residual plots, including the normal probability plot, residuals vs fitted values, and the error percentage histogram for L/D and the integral of APL. According to the figure, the residuals for each instance are calculated separately, with most values close to zero. The normal probability plots show that the fitted line slope in the integral of APL model is steeper than that of the L/D model, which results from the clustering of more instances near the zero-residual line.

In the residuals vs fitted values plots, the instances are well-distributed on both sides of the zero-residual line, indicating that the residuals do not introduce bias into the screening models. Lastly, the histograms of residuals show the frequency distribution of residuals, with many instances falling near the zero-residual axis. Overall, the results confirm that the models are sufficiently accurate for studying the main effects and two-way interactions of the inputs.

#### Main effects

The main effects analysis reveals how each individual input factor-such as pressure, temperature, Mach number, angle of attack, and winglet cant angle-affects the outputs, specifically L/D and the integral of APL, when varied independently. This type of analysis helps in understanding the direct influence of each factor on performance without considering interactions with other variables.

**Fig. 14:**
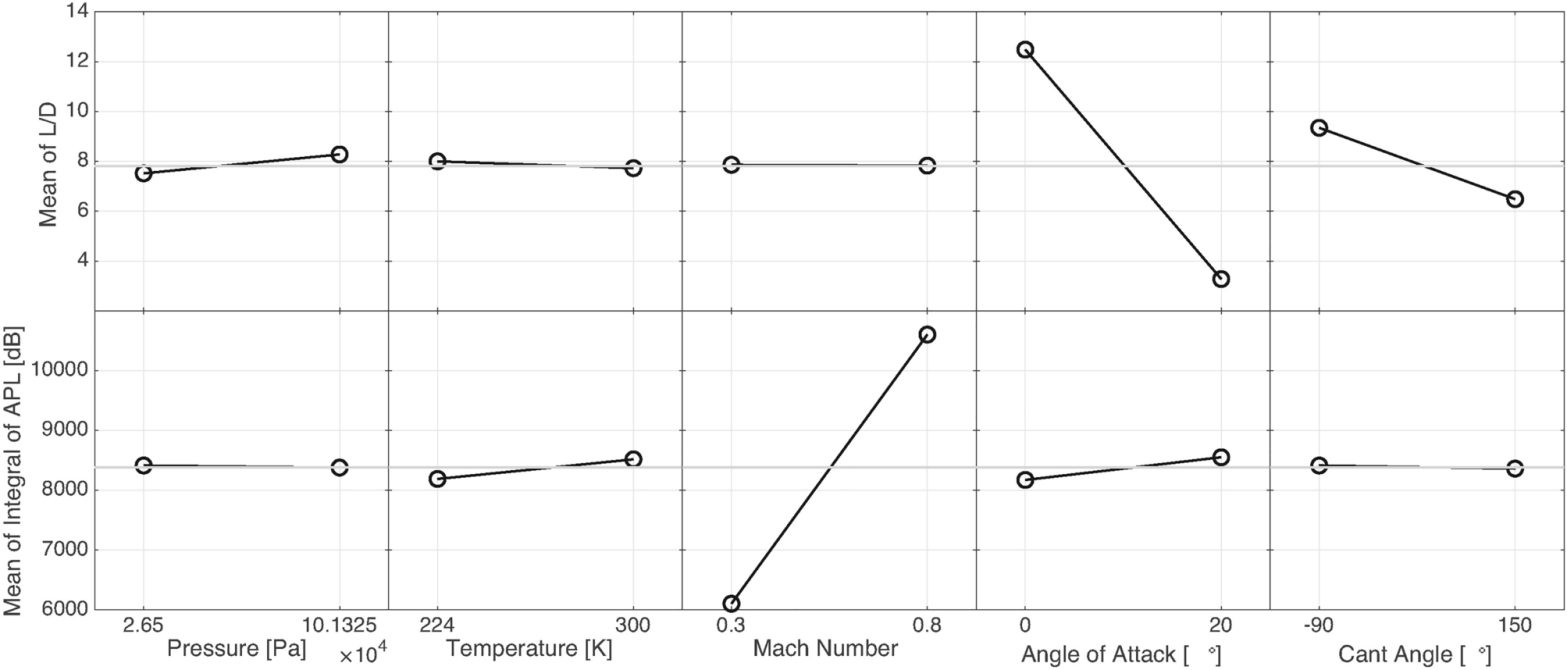
Main effects of input factors on L/D and integral of APL.

Figure 14displays the main effects for L/D, demonstrating that the most significant factors are the angle of attack and winglet cant angle. These two variables show the steepest slopes, meaning that even small changes in their values have a large impact on the aerodynamic efficiency of the wing. The angle of attack directly affects the lift generated by the wing, and changes in winglet cant angle alter the tip vortex structure, leading to variations in drag. Together, these two factors play a crucial role in optimizing aerodynamic performance.

In contrast, temperature and Mach number show much flatter slopes, indicating that their effects on L/D are relatively minor within the studied range. While temperature can influence air density and Mach number reflects flight speed, neither has a large enough effect to significantly impact the lift-to-drag ratio. Pressure, however, has a moderate impact on L/D, as it is closely tied to atmospheric conditions that can alter aerodynamic forces on the wing. This suggests that while temperature and Mach number can be considered secondary factors, pressure should still be monitored when optimizing L/D.

Figure 14 also shows the main effects for the integral of APL, where Mach number stands out as the dominant factor. The steep slope of the Mach number line indicates that changes in flight speed have a profound impact on acoustic performance, as Mach number directly influences the intensity of the noise generated by airflow over the wing. The other factors-pressure, temperature, angle of attack, and winglet cant angle-show relatively flatter slopes, implying that their effect on APL is much smaller in comparison. This highlights the fact that, while aerodynamic performance is primarily driven by angle of attack and cant angle, aeroacoustic performance is predominantly controlled by Mach number.

Interestingly, the cant angle shows a strong influence on L/D but only a minor influence on APL. This indicates that while adjusting the winglet cant angle can significantly alter aerodynamic efficiency, it has a less substantial effect on noise generation. This finding is particularly important for designers looking to balance both aerodynamic and acoustic performance, as it suggests that optimizing for L/D may not always align with minimizing APL.

#### Two-way interactions

The two-way interactions in this study provide important insights into how pairs of input factors combine to influence both the aerodynamic (L/D) and aeroacoustic (APL) outputs. These interactions can either enhance or diminish the effect of the individual factors and offer a more comprehensive understanding of the system’s behavior.

**Fig. 15:**
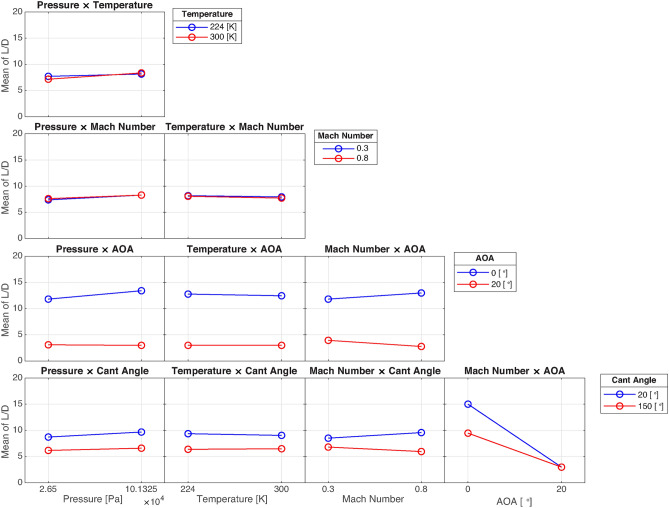
Two-way interactions of the factors on L/D.

Figure 15presents all the two-way interactions for the proposed model predicting L/D. Interactions between temperature and the other factors show the least slope, indicating that temperature, when combined with other inputs, contributes minimally to the changes in L/D. This suggests that temperature has a limited effect when it interacts with other operational or geometric factors in the studied range. However, interactions involving the angle of attack are much more pronounced. Specifically, the interaction between angle of attack and winglet cant angle shows the steepest slope, demonstrating that these two factors together significantly affect aerodynamic performance. This is expected, as both the angle of attack and cant angle directly influence the flow structure around the wing, leading to changes in lift and drag.

Pressure interactions exhibit moderate slopes, particularly when combined with the angle of attack. This indicates that while pressure doesn’t have as large of an effect as angle of attack or cant angle, it still plays a role in determining L/D, especially when paired with more dominant factors. These findings suggest that optimizing pressure conditions may be useful in fine-tuning aerodynamic performance, though it is not as crucial as controlling the winglet cant angle or angle of attack.

**Fig. 16:**
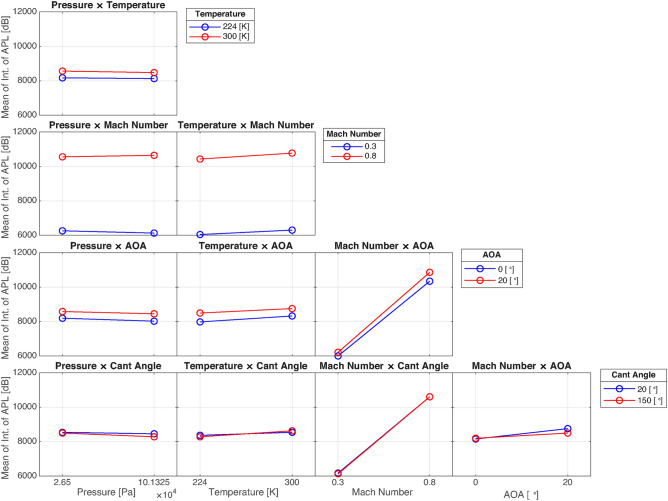
Two-way interactions of the factors on integral of APL.

Figure 16 provides the two-way interaction plots for the integral of APL, revealing more about how pairs of inputs affect aeroacoustic performance. Unlike in the case of L/D, Mach number emerges as the most dominant factor in these interactions, particularly when paired with angle of attack and winglet cant angle. The interactions between Mach number and both angle of attack and cant angle display the steepest slopes, indicating their strong combined influence on acoustic behavior. This is likely due to the fact that both Mach number and angle of attack affect the speed and direction of airflow over the wing, which in turn generates acoustic power.

Interestingly, the interaction between cant angle and other factors, particularly pressure and temperature, results in nearly coincident lines, meaning that the changes in these factors do not significantly alter the acoustic power levels when cant angle is varied. This suggests that, for controlling aeroacoustic performance, the Mach number is the most critical parameter, with other factors having a much smaller or negligible effect. In contrast, interactions between angle of attack and Mach number, and between cant angle and Mach number, provide the most substantial insights into how aeroacoustic performance can be controlled and optimized.

In summary, the two-way interaction analysis reveals the complexity of how these factors combine to influence both L/D and APL. While some factors, like temperature, play a relatively small role in these interactions, others, such as angle of attack and Mach number, emerge as dominant players, especially when paired with winglet cant angle. These results highlight the importance of focusing on these key interactions to achieve optimal performance in both aerodynamics and acoustics.

#### Comparative analysis

**Fig. 17:**
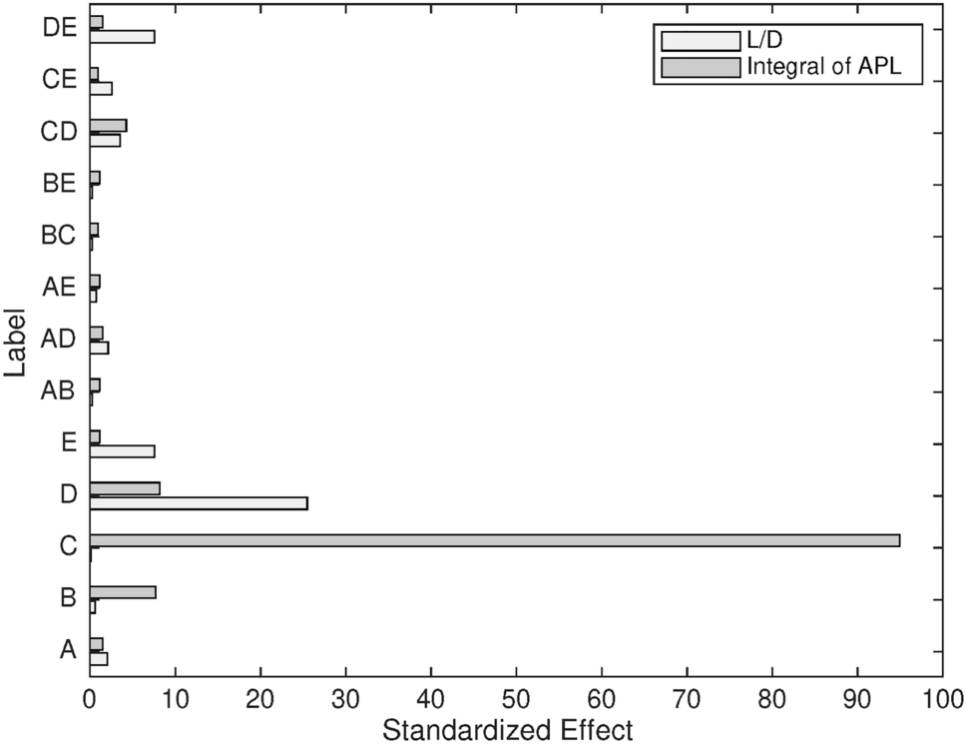
Visualization of standardized effects via Pareto chart.

Figure 17 illustrates the standardized effects of the input factors and their associated two-way interactions on both L/D and the integral of APL. Standardized effects help compare the relative impact of each factor on the outputs, providing a clear picture of which inputs are most influential.

For the integral of APL, Mach number emerges as the most influential factor, which aligns with its dominant role in determining aeroacoustic performance. As flight speed increases, the intensity of the noise generated by the airflow over the wing also increases, explaining the strong influence of Mach number on APL. The angle of attack and temperature follow as significant factors, though their effects are less pronounced compared to Mach number. This suggests that while angle of attack and temperature affect noise generation, their roles are secondary to the impact of flight speed. In terms of two-way interactions, the combination of Mach number and angle of attack is the most significant, as both parameters directly influence airflow velocity and direction, which in turn affects noise levels. The interactions between Mach number and temperature, as well as between cant angle and other factors, show relatively minor effects, indicating that these combinations contribute less to acoustic performance.

For L/D, the angle of attack is the most effective factor, followed by the winglet cant angle and pressure. The angle of attack directly influences lift generation, while changes in cant angle modify the wingtip vortex, thereby affecting drag. Pressure, while not as influential as angle of attack or cant angle, still plays a notable role in determining L/D, particularly in varying atmospheric conditions. The two-way interaction between cant angle and angle of attack is the most dominant, suggesting that these two factors together have the largest combined influence on aerodynamic efficiency. The interaction between cant angle and Mach number, as well as between cant angle and pressure, also shows a considerable effect, indicating that cant angle’s impact on L/D can be further modulated by both flight speed and atmospheric conditions.

To summarize, winglet cant angle and its two-way interactions play a crucial role in influencing L/D, particularly when combined with angle of attack and Mach number. However, when it comes to acoustic performance, cant angle can be largely neglected, as Mach number is the primary driver of APL. This distinction between the factors that influence aerodynamics and acoustics highlights the importance of targeting specific parameters based on the performance aspect being optimized-whether it is aerodynamic efficiency or noise reduction.

## Conclusion

In this work, a comparative study is conducted to assess the effect of atmospheric and operational factors on a wing’s aerodynamic and aeroacoustic performance, emphasizing winglet cant angle. Firstly, fluid physics is studied in terms of aerodynamics and acoustics by analyzing pressure coefficient and acoustic power level distribution along sections located near the wingtip and root. Subsequently, a matrix of experiments is generated utilizing full factorial design to be simulated by CFD solver for screening analysis. The obtained datasets are screened by MiniTab software to evaluate the main effects of inputs and the associated two-way interactions on L/D and integral of APL as representatives of aerodynamic and acoustic performance, respectively. Generally, the conducted analysis of the numerical datasets is concluded as follows:

- The morphology study showed that changing winglet cant angle significantly affects the pressure coefficient distribution along the span, particularly near the tip area. Winglet cant angle is able to modify the flow structure near the tip area, in which pressure distribution is altered along different sections. However, by marching to the root, the cant angle effectiveness gradually reduces, which is considered negligible, particularly in lower angles of attack. Moreover, the winglet cant angle can affect the distribution of APL on the wing surface so that its effect on the wing’s upper surface is more significant than the lower.
- The screening study illustrated that the cant angle and the associated two-way interactions significantly affect both L/D and integral of APL compared with other inputs. In terms of main effects, angle of attack, cant angle, and pressure are effective for L/D, while temperature, Mach number, and angle of attack are recognized as important factors. Regarding two-way interactions, the combinations of angle of attack with pressure and cant angle, as well as the combinations of angle of attack with Mach number and cant angle, are found to be particularly effective on L/D and integral of APL. All in all, the angle of attack is the most effective factor; however, winglet cant angle shows considerable impacts on L/D as well as the integral of APL.

In conclusion, this research revealed that winglet cant angle is an important factor in terms of the wing’s aerodynamic and acoustic performance compared to other atmospheric and operational factors. Indeed, the idea of morphing winglets is a practical manner of performance enhancement. Also, the screening analysis proved the importance of considering two-way interactions of input as well as their main effects since they may affect the performance indirectly via the former set of factors.

## Data Availability

The datasets used and/or analyzed during the current study are available from the corresponding author upon reasonable request.

## A detailed matrix of experiments

Table 7 presents the matrix of experiments designed using the full factorial design method. This approach allows for a systematic exploration of all possible combinations of input factors, enabling a thorough analysis of how each factor and their interactions influence the outputs. The factors considered in this study include pressure (A), temperature (B), Mach number (C), angle of attack (D), and winglet cant angle (E). Each of these factors is varied between lower (-) and upper (+) bounds to represent realistic operational conditions for a commercial wing operating in subsonic flow.

By employing a full factorial design, every possible combination of these five factors is evaluated. This ensures that no potential interaction between the input variables is overlooked, providing comprehensive insights into both the main effects and the two-way interactions that influence aerodynamic and acoustic performance. For instance, pressure (A) and temperature (B) are varied to simulate different atmospheric conditions, while Mach number (C) represents flight speed. Angle of attack (D) and winglet cant angle (E) are key aerodynamic parameters that directly affect lift and drag force coefficients, and wing aeroacoustic performance.

**Table 7:** Details of defined numerical simulations in the matrix of experiments

		Inputs					
Blocks	#Run	*A*	*B*	*C*	*D*	*E*	Combination
1	1	-	-	-	-	-	1
	2	+	-	-	-	-	A
	3	-	+	-	-	-	B
	4	+	+	-	-	-	AB
2	5	-	-	+	-	-	C
	6	+	-	+	-	-	AC
	7	-	+	+	-	-	BC
	8	+	+	+	-	-	ABC
3	9	-	-	-	+	-	D
	10	+	-	-	+	-	AD
	11	-	+	-	+	-	BD
	12	+	+	-	+	-	ABD
4	13	-	-	+	+	-	CD
	14	+	-	+	+	-	ACD
	15	-	+	+	+	-	BCD
	16	+	+	+	+	-	ABCD
5	17	-	-	-	-	+	E
	18	+	-	-	-	+	AE
	19	-	+	-	-	+	BE
	20	+	+	-	-	+	ABE
6	21	-	-	+	-	+	CE
	22	+	-	+	-	+	ACE
	23	-	+	+	-	+	BCE
	24	+	+	+	-	+	ABCE
7	25	-	-	-	+	+	DE
	26	+	-	-	+	+	ADE
	27	-	+	-	+	+	BDE
	28	+	+	-	+	+	ABDE
8	29	-	-	+	+	+	CDE
	30	+	-	+	+	+	ACDE
	31	-	+	+	+	+	BCDE
	32	+	+	+	+	+	ABCDE
